# Definitions of professional identity formation. A review of the literature and conceptual analysis

**DOI:** 10.3205/zma001827

**Published:** 2026-03-23

**Authors:** Moritz Schumm, Alexander Kremling, Pascal O. Berberat, Jan Schildmann, Christiane Vogel

**Affiliations:** 1 TUM Medical Education Center, Department Clinical Medicine, TUM School of Medicine and Health, Technical University of Munich, Munich, Germany; 2 Martin Luther University Halle-Wittenberg, Profile Center for Health Science, Institute for History and Ethics of Medicine, Halle (Saale), Germany

**Keywords:** professional identity formation, professionalism, medical education, definition, professional identity

## Abstract

**Aim:**

The purpose of this review is to better understand the terminology of professional identity formation (PIF). Guided by the question: “How is PIF defined?”, the article aims at delivering an overview and an analysis of so far established ways of understanding PIF focusing on explicit definitions and main conceptual contributions.

**Method:**

A systematic literature search was conducted via MEDLINE and CINAHL. The PRISMA framework was used to support a comprehensive search strategy and screening. In a first step of our conceptual analysis texts were analyzed regarding explicit definitions. In a second analytical step the main foci of the articles as well as different elements of PIF definitions were analyzed by comparing the structure and content of the definitions.

**Results:**

25 of 639 articles met the eligibility criteria. We identified three categories for a further conceptual analysis of the definitions according to the questions: (1) On which aspects of “becoming a physician” is PIF focusing? (2) What are further properties of PIF? (3) What is the result of PIF? Additionally, the main foci of the identified articles were taken into account.

The main foci of the identified articles showed diverse intentions regarding PIF. Most often “values” and “norms” were focused aspects of definitions. Regarding further properties of PIF a variety of aspects are stated unsystematically. Possible results of PIF mentioned in or in the context of definitions are most frequently to “think, act, and feel like a physician”.

**Conclusion:**

The review and analysis offer an overview over ways to define PIF and typical elements. Contextualization of these results within the publication history of PIF will further the understanding of the term’s development as well as its potential for a more comprehensive definition.

## Zusammenfassung

**Ziel:**

Zweck dieses Reviews ist es, die Terminologie der Professional identity formation (PIF) besser zu verstehen. Ausgehend von der Frage „Wie wird PIF definiert?“ will der Artikel einen Überblick und eine Analyse der bisher etablierten Ansätze zum Verständnis von PIF liefern, wobei der Schwerpunkt auf expliziten Definitionen und den wichtigsten konzeptionellen Beiträgen liegt.

**Methode:**

Es wurde eine systematische Literaturrecherche über MEDLINE und CINAHL durchgeführt. Das PRISMA-Framework wurde zur Unterstützung einer umfassenden Suchstrategie und Sichtung verwendet. In einem ersten Schritt unserer konzeptionellen Analyse wurden die Texte hinsichtlich expliziter Definitionen analysiert. In einem zweiten analytischen Schritt wurden die inhaltlichen Foki der Artikel sowie verschiedene Elemente der PIF-Definitionen durch einen Vergleich der Struktur und des Inhalts der Definitionen analysiert.

**Ergebnisse:**

25 von 639 Artikeln erfüllten die Auswahlkriterien. Wir haben drei Kategorien für eine weitere konzeptionelle Analyse der Definitionen anhand der folgenden Fragen identifiziert: (1) Auf welche Aspekte des „ärztlichen Werdens“ konzentriert sich PIF? (2) Welche weiteren Eigenschaften hat PIF? (3) Was ist das Ergebnis von PIF? Zusätzlich wurden die inhaltlichen Foki der identifizierten Artikel berücksichtigt.

Die inhaltlichen Foki der identifizierten Artikel zeigten unterschiedliche Absichten hinsichtlich PIF. Am häufigsten standen „Werte“ und „Normen“ im Mittelpunkt der Definitionen. Zu weiteren Eigenschaften von PIF werden uneinheitlich verschiedene Aspekte genannt. Mögliche Ergebnisse von PIF, die in oder im Zusammenhang mit Definitionen genannt werden, sind am häufigsten „think, act, and feel like a physician“.

**Schlussfolgerung:**

Review und Analyse bieten einen Überblick über Möglichkeiten zur Definition von PIF und typische Elemente. Die Kontextualisierung dieser Ergebnisse innerhalb der Publikationsgeschichte von PIF wird das Verständnis der Entwicklung des Begriffs sowie seines Potenzials für eine umfassendere Definition fördern.

## 1. Background

Professional identity formation (PIF) can be called a rising star when it comes to contemporary conceptions of medical education. The Carnegie Foundation’s 2010 report – often considered to have initiated a widespread discussion of the concept [1], [2], [3], [4] – stated: “professional identity formation – the development of professional values, actions, and aspirations – should be the backbone of medical education” [5]. In answer to this demand the idea has gained considerable momentum up to this day. Despite its general popularity, divergence in ways of understanding and applying PIF also makes it a multi-faced concept in which “[s]everal different, and sometimes conflicting, conceptualizations and theories of PIF populate the literature” [6]. Conceptual diversity is displayed also in a number of reviews pursuing different goals. Afshar et al., for example, aim for a unification of the field by synthesizing different approaches [7], whereas Sarraf-Yazdi et al. try to implement an alternative model for identity development [8]. Mount et al. elaborate on the consequences that different perspectives on PIF might have for the development of practical teaching [6]. In contrast to that, the following review article focuses primarily on the search and the compilation of definitions of PIF in a strict sense. Guided by the question: “How is PIF defined?”, the article aims for an overview of so far established ways of understanding PIF as well as a first comparison of specific aspects of the included articles and the identified definitions.

The idea for this review came about as part of the activities of the work undertaken by members of the committee “Professional Identity Formation” of the DACH Association for Medical Education (Gesellschaft für Medizinische Ausbildung) that, for the last two years, followed the conviction that the implementation of PIF into the teaching of medical schools also requires a better understanding of its terminology. To this end, the authors (of which four are members of the committee: MS, POB, JS, CV) developed this review to collect and analyze definitions of PIF without attempting to deliver a new definition. The latter was the subject of another working group within the above mentioned committee [9].

## 2. Methods

We carried out a systematic literature review following the guidelines of Preferred Reporting Items for Systematic Reviews and Meta-Analyses (PRISMA) [10] to identify possible definitions related to PIF. PRISMA was chosen as a framework for its singular focus on the reporting of a systematic literature review, see attachment 1 for an overview of the undertaken methodical steps. The database search was conducted from April to May 2024. Additionally to the review, a conceptual analysis of the included articles and the identified definitions was conducted according to further criteria as grounds for comparison.

### 2.1. Eligibility criteria

Inclusion and exclusion criteria were developed prior to the study selection. All decisions about inclusion and exclusion criteria were reached by consensus between the authors involved. Special attention was paid to the inclusion of general discussions of professional identity formation and the exclusion of discussions focussing on particular medical specialties. The search focused on articles written in English or German. Following the PICO [11] framework our inclusion and exclusion criteria for the search are provided in table 1 (Tab. 1).

### 2.2. Search strategy

The search for articles concentrated exclusively on the term “professional identity formation” without any further constraints (e.g. concerning publication date) to allow for a broad perspective on this term. Concerning restrictions of this search strategy see limitations section below. The two databases MEDLINE via PubMed and CINAHL via EBSCO were selected for the search. MEDLINE represents biomedicine’s most exhaustive database for literature search. CINAHL was chosen to extend the results by its focus on nursing, allied health research and healthcare.

### 2.3. Selection process

639 articles were screened by title and abstracts for eligibility against exclusion and inclusion criteria by two independent reviewers (MS, CV) during June and July of 2024. Any significant discrepancy or controversial studies were cleared after discussion and consensus by these two authors. In order to screen and assess, the studies from the search of the databases were transferred to Rayyan [https://www.rayyan.ai/] – a systematic literature review tool as well as collaboration program.

### 2.4. Charting the data/data collection process

The eligible studies were screened and analyzed and then discussed by three reviewers (MS, AK, CV) aiming at consensus on the specific definition of PIF in each article. The discussion process led to an agreement on whether there is a definition or approaches to it or if there is none. As part of reporting the results, these findings were noted and charted in a uniform and systematic way using a tabular overview (see attachment 2 ).

### 2.5. First analytical step: Extraction of definitions

We screened the texts for those sentences or paragraphs that come closest to a definition in the sense of a statement providing information on how to use the expression “professional identity formation”. We thus looked out more for sentences and paragraphs containing necessary and sufficient conditions for PIF. We understood this as a task different to e.g., the author’s main claim about PIF or their central contribution to the debate. In case no definition of PIF could be identified, we looked for the closest related defined expression like “professional identity” or “identity formation”. If we found an explicit and clear definition of a central element of the definition, we included this as well. We unified the passages as sentences of the form “PIF is defined as…” in order to improve comparison with as little changes to the original passage as possible and independent of our own opinions about a good PIF definition.

### 2.6. Second analytical step: Deeper conceptual analysis of structure and content of the extracted definitions

We then took a closer look at the content of the definitions and systematically unified the definitions without changing their meaning. We then searched for reoccurring content or types of content that allow to easily describe in which respect the definitions differ. 

## 3. Results

**Figure 1:**
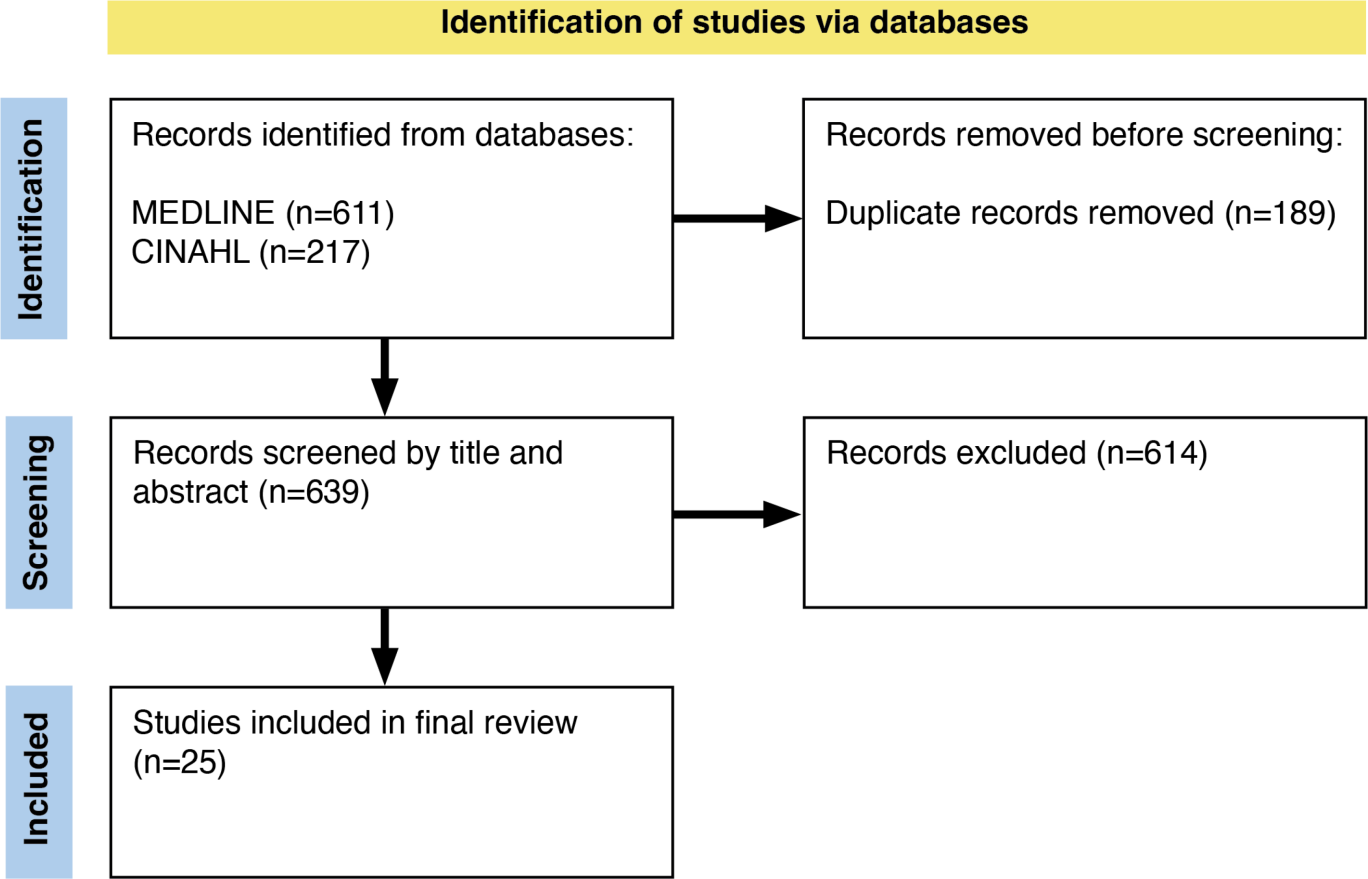
PRISMA 2020 flow diagram for new systematic reviews which included searches for databases

Our PRISMA flow diagram in figure 1 (Fig. 1) displays our search and selection results. 828 publications were identified in the database search. 25 publications were included in the review.

### 3.1. First analytical step: Extraction of definitions

Attachment 2 shows the identified definitions including a simplified version. Agreement on a definition or (a) sentence(s) closest to a definition required discussion and interpretation between the authors (MS, AK, CV). It was often not the case that PIF was clearly introduced into the text by a singular statement (e.g. [12]). In four cases we did not find a definition despite very inclusive criteria for “definition” [13], [14], [15], [16]. Often authors use a direct quote or similar phrasing with reference to other articles (especially concerning [17]). We included these references in the attachment 2 . In many cases PIF was indirectly introduced by statements already setting a focus, emphasizing a connected aspect that is of special importance to the authors. Furthermore, it was often difficult to decide about beginnings and endings of passages that introduce the concept of PIF. Sometimes it was difficult to distinguish the author’s definition from reports of a definition (that the authors might reject), or from normative claims about how professional identity should be shaped by educational measures (e.g. [7]). Moreover, the primary definendum (the defined term) was often “PIF”, sometimes “professional identity”, and sometimes it was easier to decide what the central contribution of the authors to the debate about PIF was than about their definition. Some articles introduce the term PIF into the text by connecting it to various partially overlapping other topics and/or terms rather than by a definition (e.g. [18]).

### 3.2. Second analytical step: Deeper conceptual analysis of identified definitions and articles

We identified three main reoccurring elements of the definitions according to the questions:

1. On which aspects of “becoming a physician” is PIF focusing?
2. What are further properties of PIF?
3. What is the result of PIF?

Attachment 2 shows the simplified versions of the definitions and also a color coding reflecting these elements. In the following, we describe the main foci of the included articles and then summarize the content of the identified three reoccurring elements of the definitions.

#### 3.2.1. Main foci

**Figure 2:**
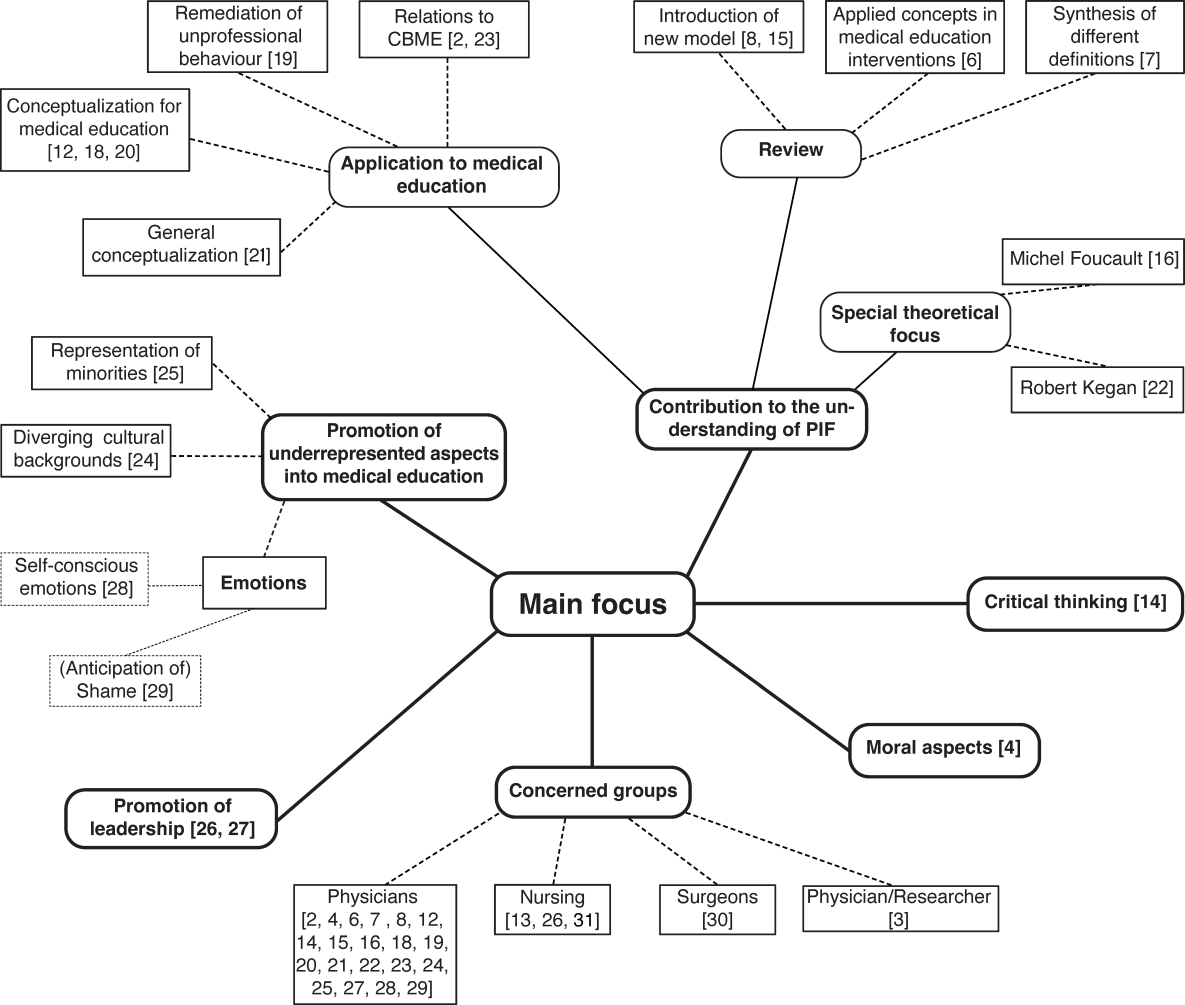
Main foci of the 25 selected articles

The 25 identified articles showed by comparison diverse backgrounds and intentions with possible influence on the identified definitions of PIF (see figure 2 (Fig. 2)).

13 of the 25 articles were identified to have the primary goal to contribute to the understanding of PIF [2], [6], [7], [8], [12], [15], [16], [18], [19], [20], [21], [22], [23]. Intentions of these articles differ widely: e.g. the review by Afshar et al. [7] is trying to deliver a synthesis of different definitions of PIF whereas the scoping review by Sarraf-Yazdi et al. [8] aims at a new model (“ring theory of personhood”) for PIF while Cruess et al. [2] and Sternszus et al. [23] try to locate PIF within competence-based medical education. Four articles see PIF as a chance to incorporate so far underrepresented aspects into medical education: Helmich et al. [24] advocate for a greater sensibility towards diverging cultural backgrounds while Volpe et al. [25] focus on the representation of minorities in medical education. Two articles promote leadership as a crucial factor of PIF [26], [27], while Bynum et al. [28] as well as Lusk [29] concentrate on the importance of specific emotions in medical training. Kim et al. [4] focus on moral aspects as crucial factors for PIF and Hafferty et al. [14] are looking at the potential for critical thinking in PIF to enable change in the current medical system. The divergence of thematic orientation can be extended by also taking the different “target groups” of the articles into account: Most articles (20) are concerned with a physician’s identity, but there are also articles highlighting the specificity of PIF for surgeons [30] and nurses [13], [26], [31] while Rosenblum et al. [3] focus on PIF for physicians also working in the field of research.

#### 3.2.2. Aspects of “becoming a physician”

A far-reaching agreement on certain terms can be discovered: “Value” is mentioned eleven times [2], [3], [6], [12], [19], [20], [23], [26], [28], [29], [30]. It is followed by “norms” [6], [19], [20], [23], [26], [29] and “characteristics” [6], [19], [20], [23], [26], [29] with each term being used six times. “Aspirations” [2], [3], [12], [30] as well as “actions”, “behaviors”, “knowledge” and “skills” [6], [12], [28] also rank high. We would like to emphasize that every definition remains neutral about the exact nature of the values, norms etc. and there is no attempt to spell out desirable values/norms. In this sense, PIF is unanimously defined neutrally.[26]

#### 3.2.3. Further properties of PIF

The 25 selected articles share an understanding of PIF as a process, but differ in the further description. Some formulations, for example, focus on PIF as a “continuous” process [7], [12] to indicate its “ongoing” [6] and “career-long” [25] qualities. On other instances the “dynamic” [12], [18], [22], [28], “non-linear” [7], [8], “fluid” [8] and “complex” [4], [6], [8], [12] nature of PIF is highlighted – and with it the variability of the process.

At the same time, there is a widely shared understanding to be found when looking at the structural parameters on which many definitions rely. Described by Jarvis-Selinger et al. [17] when speaking of a “process that happens simultaneously on two levels: (1) at the level of the individual […] and (2) at the collective level”, this “psychosocial” [4], [7] interplay is informative for many articles. Talking about PIF as socialization [3], [4], [12], [20], [23], [31] with the goal of becoming a part of the “medical community of practice” [18] or establishing a “sense of affiliation” [3] might lean more on the side of a “socially constructed” [12] process. In descriptions of the process as “development” [2], [30] or “developmental” [4], [12], [20], [23], [31] as “transformation” [8], [21] or “transformative” [6], [12], the focus “seems to fall more acutely on the individual” [25]. But both sides are embedded in a structure of codependency with articles [4], [20], [23], [31] explicitly relying on Jarvis-Selinger et al. [17] or using a similar model of understanding as Volpe et al.’s [25] metaphor of PIF as a double helix.

Agreement on this rather general structure can – yet again – be confronted with a closer look at details. The question, for example, how much agency is given to the individual health professional in the process of PIF is elaborated on in different ways. Some expressions seem to suggest a passive formation of the health professional as being affected by “multiple factors inside and outside of the educational system” [18]. Other phrasings use a more active vocabulary instead. When PIF is described as being “achieved” [18], [19], [20] or when “individuals negotiate” [6], [23], “navigate” [8] or “merge” [6] specific aspects of PIF, a creative as well as constructive role is given to health professionals.

#### 3.2.4. Results of PIF

The articles often refer to „think, act, and feel like a physician“ when it comes to describing a result of the process of PIF. In its original articulation it has to be ascribed to Merton [32], but has been popularized by Cruess et al. [2], [18], [20]. The quote or similar formulations like “thinking, acting, and feeling like a physician” or “thinking, acting, and feeling like a nurse” are featured – besides Cruess et al. [20] itself – directly in five of the identified definitions [6], [19], [23], [26], [29] and an extra of four times within the overall context of the articles [12], [15], [16], [31].

## 4. Discussion

### 4.1. Interpretation of results 

In this systematic review, we aimed to discover how professional identity formation is defined in healthcare profession literature. The 25 studies we closely reviewed in terms of their definition of PIF demonstrated insights into the terminological development of PIF as well as its functions for different stakeholders. Especially the look on the main foci of the articles (see figure 2 (Fig. 2)) shows that PIF is situated at a crossroad with different perspectives and intentions informing the understanding and role of PIF within medical education. But within this diverse field of perspectives and interests first forms of consolidation can be identified. 

First, the description of PIF as a “process” as well as the characterization of PIF as an interplay of “individual” and “collective” aspects [17] both represent a widely shared agreement on a structural model. Even if further characterization is following different routes, this structure seems to constitute a form of blueprint and common ground for the understanding of PIF.

Second, the shared use of specific phrases – even if not applying to strict criteria for definition – hints in the direction of common-sense or intuitive understanding. When it comes to the repeated use of the terms “values”, “norms”, “characteristics” as well as “actions” and “aspirations” the reason may be found in two quotes: The 2010’s Carnegie foundation report entertains the formulation “values, actions, and aspirations” [5] while Cruess et al. [20] speak of “norms, values, and characteristics”. Both sources play important roles in the development of PIF with the Carnegie foundation as a historical starting point and Cruess et al. as the “most commonly referenced authors for defining PIF in the medical education literature” [4] – especially as the first to deliver a comprehensive anthology on the subject [33]. In the case of the phrase “think, act, and feel like a physician”, it is interesting to note that only four articles refer to Merton [32] directly [2], [16], [18], [20] and three [12], [19], [23] offer Cruess et al. [18], [20] as a source. The rest comes with no reference. This does not seem to be an error, but the consequence of a repeating of an expression that has become a catchphrase and chiffre for an intuitive comprehension of PIF. Just like Latour was describing the generation of a scientific fact as a process by which a claim loses its authorship [34], [35], “values”, “norms”, etc. as well as “thinking, acting, and feeling like a physician” seem to become – even if yet in a general form – a commonplace for the understanding of PIF. The neutrality concerning specific values/norms etc. in the definitions highlights the importance of specifying these crucial aspects when it comes to developing outlines of an educational PIF program – of guiding future health care professionals towards certain values/norms. The definitions do not settle this – from the educational perspective crucial – debate. 

### 4.2. Limitations

The review was targeting a reliable number of articles to search for definitions of PIF. However, searching other databases such as the “Core bibliographic databases” and “additional databases” as, for example, recommended by the BEME cooperation [36], would have delivered a more extensive stock of articles with possible extra findings for review.

The search for articles concentrated exclusively on the term “professional identity formation” and was strictly limited to these findings. Articles using the searched term only partly or using alternative terms are not represented in the corpus.

As described in the results section, retrieving definitions from the selected 25 articles by discussion to reach consensus required a serious amount of interpretation and compromise. Although using a strict guideline for the inclusion of definitions other formulations were also accepted when no other defining element could be identified. This accounts for passages that (partly) do not fulfill all formal requirements of a definition and/or passages that were not concerned with defining PIF as a whole (e.g. “professional identity is …” [23]) and/or passages that used other terms than PIF (e.g. “professional socialization” [7]).

## 5. Conclusion

Concerning the findings of this article, PIF is described as a developmental process but the definition of the process of PIF (still) seems to be by itself a process. This can be described as a lack of clarity with regard to terminological determination. Future research on the definition of PIF (in various contexts/disciplines) may in this perspective improve the understanding of this manifold process. A desideratum towards this article may be a first step to start a discussion about good and useful definitions of PIF.

A systematic collection and structural description of the content of existing definitions provides a starting point for a reflected and conscious choice of a definition. This is of special importance for the applicability of PIF in medical education, because deciding for one and against another form of understanding might change the way PIF is implemented into medical curricula. Tending, for example, towards a focus on the individual and her/his own agency in this process would describe PIF to a large extent as a personal choice. Teaching would accordingly lean on voluntary formats providing assistance in this development wherever it is wanted. Leaning, in contrast, towards the socialization and its specific “norms, values, and characteristics” [20] is advocating for a more straightforward approach by teaching these specifics to provide students with the respective insights into the profession. In this case, implementation would move closer to the concept of professionalism [2] and its conviction to the general measurability and also testability of PIF. Recognizing not only professional experiences, but also the whole spectrum of events surrounding the development of an individual might transcend the horizon of medicine and could risk the concept’s general applicability for medical education.

Nevertheless, the terminological openness can also be appreciated as a dynamic that is providing the concept of PIF with a necessary flexibility and inclusiveness to make its point. With medical education being itself a highly diversified field with a lot of diverse perspectives involved, PIF’s becoming might be without a final and distinct consolidation, but with the potential for a growing support from different stakeholders and by that also with the ability to make an impact.

## Acknowledgements

Theresa Schneider (Institute for History and Ethics of Medicine, Profile Center for Health Science, Martin Luther University Halle-Wittenberg) and Simon Müller (TUM Medical Education Center, Department Clinical Medicine, TUM School of Medicine and Health, Technical University of Munich) – both student assistants – helped during the screening process of the search results. We would like to thank them for their support.

## Authors’ ORCIDs

- Moritz Schumm: [0009-0008-2663-7815]
- Alexander Kremling: [0000-0002-2674-2497]
- Pascal O. Berberat: [0000-0001-5022-5265]
- Jan Schildmann: [0000-0002-5755-7630]

## Competing interests

The authors declare that they have no competing interests. 

## Supplementary Material

PRISMA 2020 Checklist

List of identified definitions

## Figures and Tables

**Table 1:**
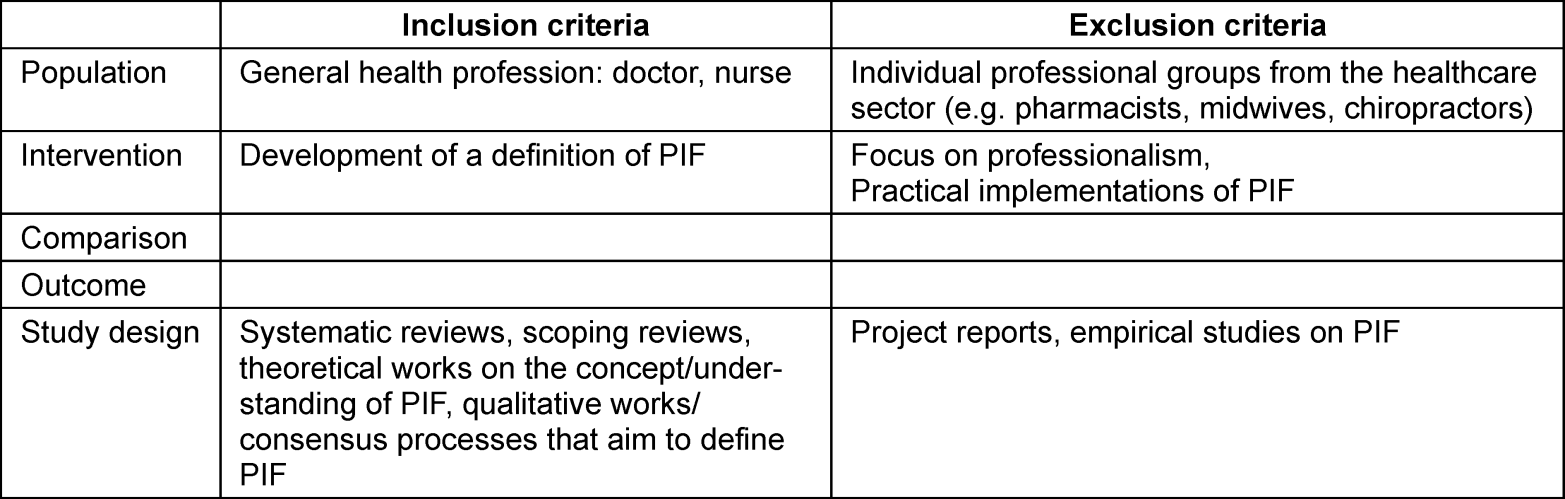
Inclusion and exclusion criteria for study selection
