# Supplementary material for: Definitions of professional identity formation. A review of the literature and conceptual analysis
Source: GMS J Med Educ. 2026 Mar 23;43(3):Doc33. doi: 10.3205/zma001827 (PMC13054814; doi:10.3205/zma001827)
Supplement: List of identified definitions [file JME-43-33-s-002.pdf]

Attachment 2: List of identified definitions

Attachment 2 to Schumm M, Kremling A, Berberat PO, Schildmann J, Vogel C. Definitions of professional identity formation. A review of the literature and conceptual analysis. GMS J Med Educ. 2026;43(3):Doc33. DOI: 10.3205/zma001827

References	Extracted definitions	Simplified definitions
Afshar L et al. 2021 [7]	“According to this definition, professional socialization is a non-linear, continuous, interactive, personal and psychosocial process that is formed through the internalization of the specific culture of the professional community and its main outcome is the formation of professional identity.” (212; ref. Sadeghi Avval Shahr H et al. 2019 [37])	Professional identity formation is defined as the main outcome of professional socialization. Professional socialization is non-linear, continuous, interactive, personal and psychosocial process that is formed through the internalization of the specific culture of the professional communit and its main outcome is the formation of professional identity.
Barnhoorn PC et al. 2019 [19]	“PIF can be defined as the process of forming »a representation of self, achieved in stages over time during which the characteristics, values, and norms of the medical profession are internalized, resulting in an individual thinking, acting [,] and feeling like a physician«.” (304; cit. Cruess RL et al. 2014 [20])	Professional identity formation is defined as the process of forming a repr - sentation of self, achieved in stages over time during which the characteris-tics, values, and norms of the medical profession are internalized, resulting in an individual thinking, acting and feeling like a physician.
Bynum WE 4th et al. 2018 [28]	“Professional identity formation is a dynamic process by which the knowledge, skills, values, and behaviors of a physician are integrated with a learner’s personal identity.” (877; ref. Holden MD et al. 2015 [38])	Professional identity formation is defined as a dynamic process by which the knowledge, skills, values, and behaviors of a physician are integrated with a learner’s personal identity.
Cruess RL et al. 2016 [2]	“The Carnegie Foundation report on the future of medical education brought the issue to the forefront. Its authors stated that »professional identity formation—the development of professional values, actions, and aspirations—should be the backbone of medical education.” (181, cit. Cooke M et al. 2010 [5])	Professional identity formation is defined as the development of professional values, actions, and aspirations.
Cruess RL et al. 2015 [18]	“Identity Formation is a dynamic process achieved through socialization; it results in individuals joining the medical community of practice. Multiple factors within and outside of the educational system affect the formation of an individual’s professional identity.” (718)	Professional identity formation is defined as the dynamic process achieved through socialization, resulting in individuals joining the medical community of practice, influenced and a fected by multiple factors within and outside of the educational system.
Cruess RL et al. 2014 [20]	“In attempting to provide a definition more specific to physicians, we propose the following: »physician’s identity is a representation of self, achieved in stages over time during which the characteristics, values, and norms of the medical profession are internalized, resulting in an individual thinking, acting, and feeling like a physician.” (1447)	Professional identity formation is defined as the socialization process by which a person learns to function within a particular society.
	“Jarvis-Selinger et al have provided a clear definition of the process of identity formation: »an adaptive developmental process that happens simultaneously at two levels: (1) at the level of the individual, which involves the psychological development of the person and (2) at the collective level, which involves the socialization of the person into appropriate roles and forms of participation in the community’s work.” (1448; cit. Jarvis-Selinger S et al. 2012 [17])	Professional identity formation is defined as the adaptive developmental process happening at (1) the individual level, which involves the psychological development of the person and (2) the collective level, which involves the socialization into appropriate roles and forms of participation in the community’s work.
Gkiousias V 2021 [30]	“Professional identity includes the development of professional aspirations, values, and actions, as a result of personal reflections and interactions with one’s workplace and the wider society.” (1; ref. Cruess RL et al. 2016 [2], Jarvis-Selinger S et al. 2012 [17])	Professional identity formation is is defin d as the development of professio-nal aspirations, values, and actions, as a result of personal reflections and interactions with one’s workplace and the wider society.
Goodolf DM et al. 2021 [31]	“Professional identity formation has previously been defined as, »[a]n adaptive developmental process that happens simultaneously at two levels: (1) at the level of the individual, which involves the psychological development of the person and (2) at the collective level, which involves the socialization of the person into appropriate roles and forms of participation in the community’s work.«” (493; cit. Jarvis-Selinger S et al. 2012 [17])	Professional identity formation is defined as the adaptive developmental process happening at (1) the individual level, which involves the psychological development of the person and (2) the collective level, which involves the socialization into appropriate roles and forms of participation in the community’s work.
Goodolf DM 2018 [13]	No definition found	
Hafferty FW et al. 2016 [14]	No definition found	
Helmich E et al. 2017 [24]	“Professional identity formation is not only about learning to display appropriate professional behavior but is fundamentally about developing a new, professional identity as a physician.” (61)	Professional identity formation is defined as developing a new, professional identity as a physician and is not only about learning to display appropriate professional behavior.
Holden M et al. 2012 [21]	“More recently, authors have begun to discuss professional identity formation (PIF), distinguishing it as the foundational process one experiences during the transformation from lay person to physician.” (246; ref. Inui TS 2003 [39])	Professional identity formation is the foundational process one experiences during the transformation from lay person to physician.

References	Extracted definitions	Simplified definitions
Kim DT et al. 2024 [4]	"…the process by which medical learners are socialized into the profession …" (399)	Professional identity formation is defined as the process by which medical learners are socialized into the profession.
	"We […] propose to describe medical learners as internalizing the social norms of medicine not only on psychosocial terms but also as moral agents who actively self-reflectively aspire to flourish as good physicians." (400)	Professional identity formation is defined as the process of internalizing the social norms of medicine not only on psychosocial terms but also as moral agents who actively self-reflectively aspire to flourish as good physicia .
	"An early definition of PIF in the medical education literature, for example, describes an »adaptive, developmental process« that involves both an individual's »psychological development« and, at the collective level, »the socialization of the person into appropriate roles and forms of participation in the community's work.«" (400; Jarvis-Selinger et al. 2012 [17])	Professional identity formation is defined as the adaptive developmental process happening at (1) the individual level, which involves the psychological development of the person and (2) the collective level, which involves the socialization into appropriate roles and forms of participation in the community's work.
Lewin LO et al. 2019 [22]	"The formation of a physician's professional identity is a dynamic process shaped by and intertwined with the development of that person's larger adult identity. Both are influenced by many internal and external factors and can be viewed through a variety of complex theoretical perspectives." (1299; ref. Vignoles VL et al. 2011 [40])	Professional identity formation is defined as a dynamic process shaped by and intertwined with the development of that person's larger adult identity, both are influenced by many internal and external factors and can be viewed through a variety of complex theoretical perspectives.
Lindell Joseph M et al. 2021 [26]	"Professional identity in nursing (PIN) is defined as »a sense of oneself, in relationship with others, that is influenced by characteristics, norms and values of the nursing discipline, resulting in an individual thinking, acting and feeling like a nurse." (27f; cit. Benner P et al. 2010 [41], Fitzgerald A 2020 [42])	Professional identity formation (of nurses) is the process of developing a sense of oneself, in relationship with others, that is influenced by characteristics, norms and values of the nursing discipline, resulting in an individual thinking, acting and feeling like a nurse.
Lusk P 2021 [29]	"Professional identity in medicine has been defined as a ›representation of self, achieved in stages over time during which the characteristics, values, and norms of the medical profession are internalised‹. […] The broad theory of professional identity formation aims to explore the process through which professional values become rooted in one's identity – how one begins to feel and act like a physician." (275; cit. Cruess RL et al. 2014 [20])	Professional identity formation is defined as the process of developing a r -presentation of self achieved in stages over time during which the characteristics, values, and norms of the medical profession are internalised, of medical professional values becoming rooted in one's identity, of beginning to feel and act like a physician.
Maile E et al. 2019 [27]	"All individuals hold multiple identities, and for doctors, one of these is their MPI [medical professional identity], formed through the process of MPIF [medical professional identity formation], which consists of three overlapping domains: professionalism, identity development and formation." (587; ref. Holden M et al. 2012 [21])	Professional Identity Formation is defined as the process which consists of three overlapping domains: professionalism, identity development and formation.
Mount GR et al. 2022 [6]	"Professional identity formation (PIF) can be defined as the integration of the knowledge, skills, values, and behaviors of a profession with one's preexisting identity and values." (S96)	Professional identity formation is defined as the integration of the knowledge, skills, values, and behaviors of a profession with one's preexisting identity and values.
	"PIF is understood as the complex, multidimensional, ongoing, and transformative process through which individuals negotiate between or merge their preexisting knowledge, skills, values, and behaviors with those they perceive as embedded in their chosen career." (S96; ref. Wald HS 2015 [12], Holden MD et al. 2015 [38])	Professional identity formation is defined as complex, multidimensional, ongoing, and transformative process through which individuals negotiate between or merge their preexisting knowledge, skills, values, and behaviors with those they perceive as embedded in their chosen career.
	"From this orientation, PIF is the process through which the individual develops and maintains the desired values, attitudes, and attributes of a physician." (S96; ref. Monrouxe LV 2015 [43])	Professional identity formation is defined as the process through which the individual develops and maintains the desired values, attitudes, and attributes of a physician.
	"Overall, the manuscripts in the corpus acknowledged—even if only implicitly—PIF as a process where internalization or integration of characteristics, values, and norms leads to a representation of self, with individuals ultimately beginning to think, act, and feel like a physician." (99)	Professional identity formation is defined as the process where internalization or integration of characteristics, values, and norms leads to a representation of self, with individuals ultimately beginning to think, act, and feel like a physician.
Rosenblum ND et al.2016 [3]	"The 2010 Carnegie Report on Educating Physicians, which called for a focus on professional identity formation in medical education, defined professional identity as a composite of the values, beliefs, sense of affiliation, aspirations, and synchrony with the norms of the medical profession." (1612; ref. Cooke 2010 [5])	Professional identity formation is defined as the process of developing a composite of the values, beliefs, sense of affiliation, aspirations, and sy -chrony with the norms of the medical profession.
	"Professional identity forms through a socialization process in which a new professional identity is integrated with one's personal identity." (1613; ref. Monrouxe LV 2010 [44])	Professional identity formation is defined as the socialization process of integrating a new professional identity with one's personal identity.

References	Extracted definitions	Simplified definitions
Sarraf-Yazdi S et al. 2021 [8]	"Holden et al. describe professional identity formation (PIF) »as the foundational process one experiences during the transformation from lay person to physician«." (3512; cit. Holden M et al. 2012 [21])	Professional identity formation is defined as the foundational process one experiences during the transformation from lay person to physician.
	"PIF is a complex, non-linear and fluid process through which medical students navigate competing influences between their professional roles and personal lives, and iteratively construct and deconstruct evolving views of the self." (3518)	Professional identity formation is defined as the complex, non-linear and fluid process through which medical students navigate competing influences between their professional roles and personal lives, and iteratively construct and deconstruct evolving views of the self.
Sarraf-Yazdi S et al. 2024 [15]	No definition found	
Schrewe B et al. 2022 [16]	No definition found	
Sternszus R et al. 2023 [23]	"Professional identity is a representation of self whereby the characteristics, values, and norms of the medical profession are internalized, negotiated, and/or adapted, resulting in thinking, acting, and feeling like a physician. It is reflected both in how others perceive the learner and how the learner perceives themselves, and it is heavily informed by one's personal identities. PIF is the developmental process whereby this professional identity emerges over time, through socialization of an individual into a community of practice." (508; ref. Jarvis-Selinger S et al. 2012 [17], Cruess RL et al. 2014 [20], Cruess RL et al. 2015 [18], Lave J et al. 1991 [45])	Professional identity formation is defined as the developmental process whereby a representation of the self emerges over time through socialization of an individual into a community of practice whereby the characteristics, values, and norms of the medical profession are internalized, negotiated, and/or adapted, resulting in thinking, acting, and feeling like a physician.
Volpe RL et al. 2019 [25]	"Professional identity formation (PIF) in medicine focuses attention on the career-long process of becoming a clinician. PIF can be thought of as a double helix: the individual and the profession form parallel strands that become intertwined." (120; ref. Irby DM et al. 2016 [46], Cruess RL et al. 2015 [18]	Professional identity formation is defined as the career-long process of becoming a physician as a process of the parallel individual and the profession strands becoming intertwined.
Wald HS 2015 [12]	"PIF is an active, developmental process which is dynamic and constructive and is an essential complement to competency-based education. PIF encompasses development of professional values, moral principles, actions, aspirations, and ongoing self-reflection on the identity of the individual and is described ultimately as a complex structure that an individual uses to link motivations and competencies to a chosen career role." (701; ref. Holden MD et al. 2015 [38], Holden M et al. 2012 [21], Wilson I et al. 2013 [47])	Professional identity formation is defined as a developmental process that is dynamic, constructive and an essential complement to competency based education, encompassing the development of professional values, moral principles, actions, aspirations, and ongoing self-reflection on the identity of the individual. It is defined as a complex structure that an individual uses to link motivations and competencies to a chosen career role.
	"[PIF] is the transformative journey through which one integrates the knowledge, skills, values, and behaviors of a competent, humanistic physician with one's own unique identity and core values. This continuous process fosters personal and professional growth through mentorship, self-reflection, and experiences that affirm the best practices, traditions, and ethics of the medical profession." (702; cit. Holden MD et al. 2015 [38])	Professional identity formation is defined as the transformative journey through which one integrates the knowledge, skills, values, and behaviors of a competent, humanistic physician with one's own unique identity and core values. It is defined as the continuous process that fosters personal and professional growth through mentorship, self-reflection, and experiences that affirm the best practices, traditions, and ethics of the medical profession.

Colour scheme: Blue: Aspects of "becoming a physician", Red: Further properties of PIF, Green: Results of PIF

Full references can be found in the main article.
